# Supplementary material for: Comprehensive Genome and Plasmidome Analysis of Antimicrobial Resistant Bacteria in Wastewater Treatment Plant Effluent of Tokyo
Source: Antibiotics (Basel). 2022 Sep 21;11(10):1283. doi: 10.3390/antibiotics11101283 (PMC9598598; doi:10.3390/antibiotics11101283)

Fig. S1

Flowchart for verification of strain clonality using comparative genomic analysis

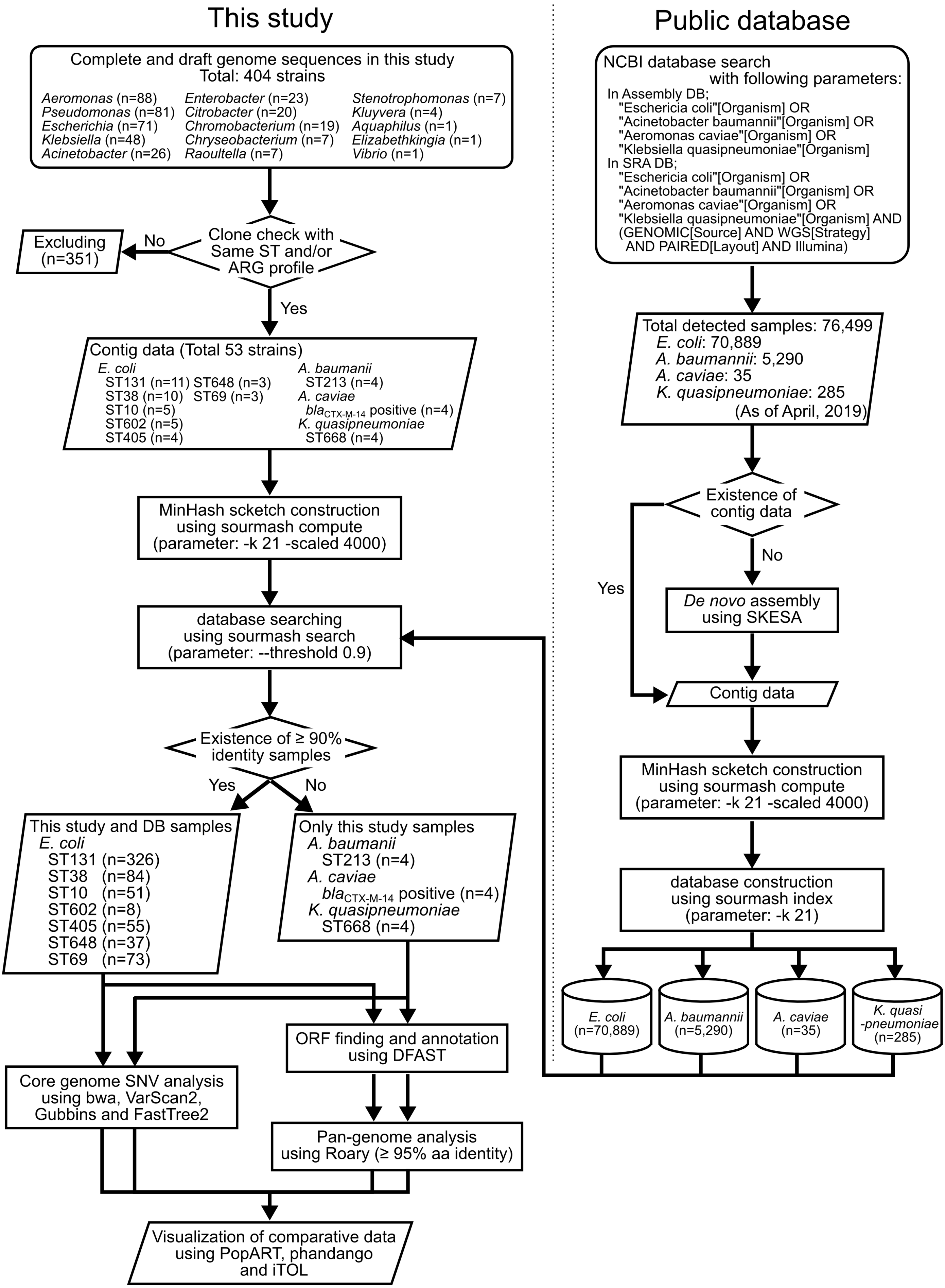

Fig. S2

Flowchart for plasmidome analysis

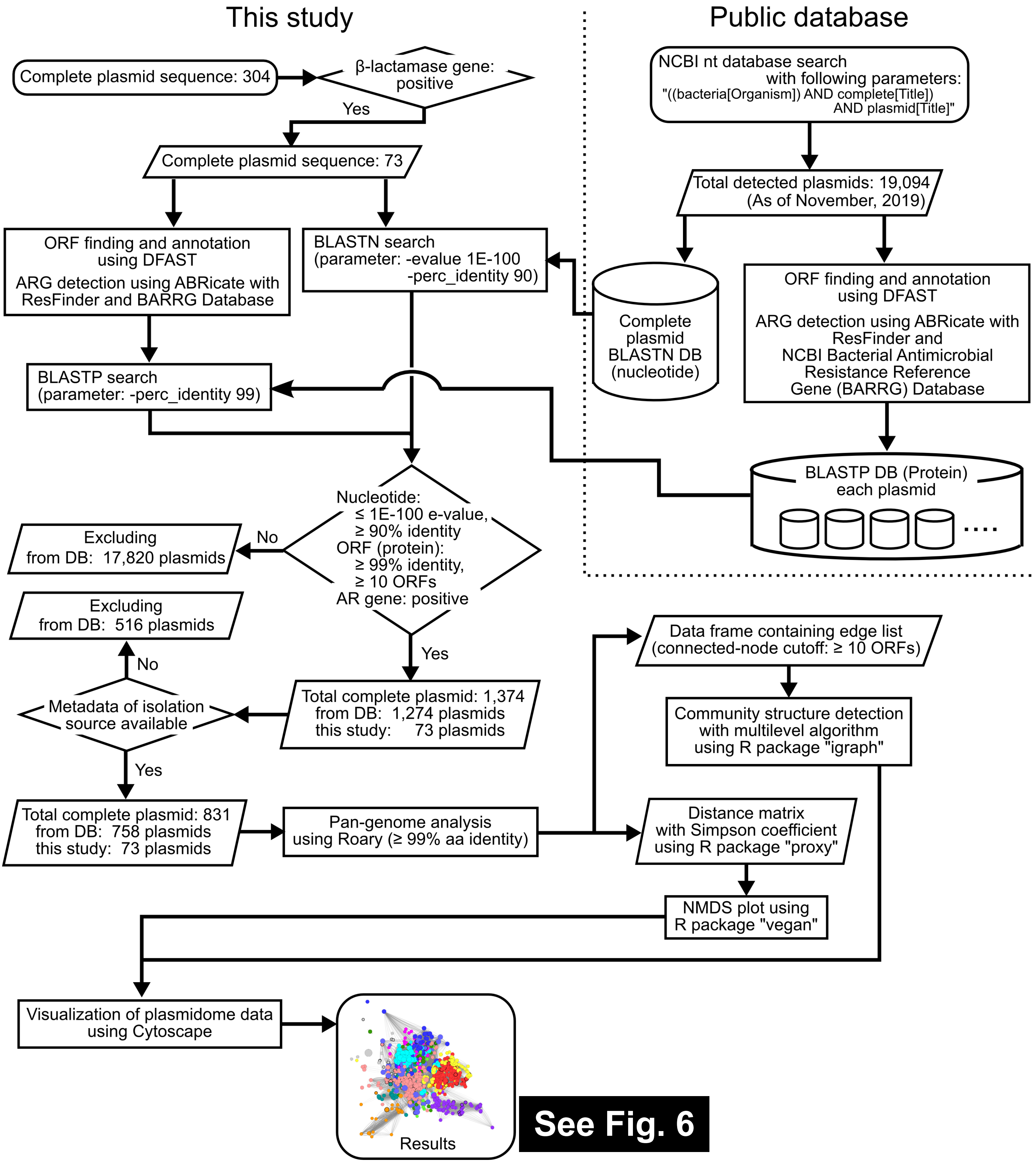

Supplement: Supplementary file 1 [file antibiotics-11-01283-s001.zip › Supplement-Figures.pdf]
